# On-line analyte preconcentration with atomic spectrometric detection

**DOI:** 10.1155/S1463924691000445

**Published:** 1991

**Authors:** L. Ebdon, A. S. Fisher, S. J. Hill, P. J. Worsfold

**Affiliations:** Plymouth Analytical Chemistry Research Unit Department of Environmental Sciences Polytechnic South West Drake Circus, Devon Plymouth PL4 8AA UK

## Abstract

Pre-concentration of analytes, or matrix removal to overcome interferences using mini- or micro-columns of exchange media prior to atomic spectrometric detection is becoming increasingly more common. This paper is a review of some of the more recent applications of chelating, ion exchange and other resins and gels that have been used to accomplish this.


Journal of Automatic Chemistry, Vol. 13, No. 6 (November–December 1991), pp. 281–286

### On-line analyte preconcentration with atomic spectrometric detection

**L. Ebdon, A. S. Fisher, S. J. Hill and P. J. Worsfold**
*Plymouth Analytical Chemistry Research Unit, Department of Environmental Sciences, Polytechnic South West, Drake Circus, Plymouth, Devon PL4 8AA, UK*

*Pre-concentration of analytes, or matrix removal to overcome interferences using mini- or micro-columns of exchange media prior to atomic spectrometric detection is becoming increasingly more common. This paper is a review of some of the more recent applications of chelating, ion exchange and other resins and gels that have been used to accomplish this.*

### Introduction

Many atomic spectrometric methods of analysis, including electrothermal atomic absorption spectrometry (ETAAS) and inductively coupled plasma atomic emission spectrometry (ICP-AES), are prone to matrix interferences. Even inductively coupled plasma mass spectrometry (ICP-MS), which is generally regarded as being relatively free from interferences, suffers from polyatomic ion interferences, especially for the determination of first row transition metals. These interferences are summarized in table 1.

Ion exchange and chelation exchange are increasingly being used for pre-concentration and/or matrix removal prior to atomic spectrometric detection. They provide a relatively cheap, robust and repeatable method of pretreatment, both on- and off-line that is easily automated, using either flow injection (FI) or high pressure liquid chromatographic (LC) techniques. Mini-columns of exchange material with dimensions of 2.0 × 0.2 cm or less provide sufficient exchange capacity for many applications. This paper provides an overview of on-line techniques for pre-concentration and/or matrix removal with particular emphasis on atomic spectrometric detection.

*Table 1. Polyatomic ion interferences of importance in food.*

| | Analysis by ICP-MS/96 | | |
|---|---|---|---|
| Mass | Element | % Abundance | Interfering ions |
| 47 | Ti | 07·32 | $PO^+$ |
| 48 | Ti | 73·98 | $^{32}SO^+$, $POH^+$ |
| 51 | V | 99·76 | $^{35}ClO^+$, $^{34}SOH^+$ |
| 52 | Cr | 83·76 | $^{40}Ar^{12}C^+$, $^{35}ClOH^+$ |
| 53 | Cr | 09·51 | $^{37}ClO^+$ |
| 54 | Fe | 05·82 | $^{40}ArN^+$, $^{37}ClOH^+$ |
| 54 | Cr | 02·38 | $ArN^+$, $ClOH^+$ |
| 55 | Mn | 100 | $^{40}ArNH^+$ |
| 56 | Fe | 91·66 | $^{40}ArO^+$ |
| 63 | Cu | 69·01 | $^{40}ArNa^+$ |
| 64 | Zn | 48·89 | $HPO_2^+$, $^{32}SO_2^+$, $^{32}S_2^+$, $^{63}CuH^+$ |
| 65 | Cu | 30·09 | $H^{32}SO_2^+$ |
| 69 | Ga | 60·16 | $^{37}ClO_2^+$ |
| 75 | As | 100 | $^{40}Ar^{35}Cl^+$ |
| 76 | Se | 09·02 | $^{36}Ar^{40}Ar^+$ |
| 77 | Se | 07·58 | $^{40}Ar^{37}Cl^+$ |
| 78 | Se | 23·52 | $^{40}Ar^{38}Ar^+$ |
| 79 | Br | 50·54 | $^{38}Ar^{40}ArH^+$ |
| 80 | Se | 49·82 | $^{40}Ar^{40}Ar^+$ |

### Chelation

Many exchange media have been developed, including anion, cation and chelation exchange resins. Chelation exchange is one of the most common forms of sample pretreatment, which can be performed either on-line or off-line. If performed on-line it offers numerous advantages, including removal of the matrix/interferents and pre-concentration with a reduced risk of contamination. The most common chelating resin is Chelex-100, which contains iminodiacetate (IDA) functional groups. These groups readily chelate transition metal ions and other cations, for example lead (II). The IDA group is also present in a number of other chelating resins, for example Muromac-A1, Metpac CC1 and Dowex A1. The first workers to report the use of Chelex-100 are acknowledged to be Riley and Taylor [1]. Several other chelating agents exist, including 8-quinolinol and more specialized resins that chelate specific ions. A list of recent applications of chelating resins is given in table 2.

The first on-line pre-concentration with atomic spectrometric detection was reported by Olsen *et al.* [8] who used Chelex-100 to pre-concentrate metals from seawater. One problem with the use of Chelex-100 is the swelling and contracting of the resin associated with changes in its ionic form. Other resins with the same functional group, for example Metpac CC1, have been reported not to suffer this disadvantage [41]. The advantage of chelating resins is that they are fairly selective for transition metals, with alkali and alkaline earth metals being easily eluted with an ammonium acetate buffer. The analyte is then eluted with dilute nitric acid.

Chelation may also be used to decrease interferences in ICP-mass spectrometry, for example metal oxides of titanium and molybdenum interfere with copper, zinc and cadmium determinations. By complexing the titanium and molybdenum with N-methylfurohydroxamic acid they may be retained on a Hamilton PRP-1 column, thereby removing the interference [42].

0142–0453/91 $3.00 © 1991 Taylor & Francis Ltd.

*Table 2. Some applications of chelating resins.*

| Analyte | Exchange medium | Matrix | Comments | Reference |
|---|---|---|---|---|
| V | Chelex-100 | Biological matrices | Chemical purification | [2] |
| Ba, Be, Cd, Co, Cu, Mn, Ni & Pb | Chelex-100 | — | Microcolumns and FI-ICP | [3] |
| Rare Earths | Chelex-100 | Uranium | Detection by NAA | [4] |
| Transition metals & Pb | Chelex-100 | Seawater | Detection by GFAAS | [5] |
| Cd, Cu, Mn, Zn & Pb | Chelex-100 | Water | IC-FAAS online elution with cysteine | [6] |
| Cd, Cu, Pb & Zn | Chelex-100 | — | FAAS detection | [7] |
| Cd, Cu, Pb & Zn | Chelex-100 | Seawater | FI-AAS, microcolumn | [8] |
| Transition metals & Pb | Chelex-100 | Seawater | GFAAS detection | [9] |
| Cd, Pb, Ni, Cu & Zn | Chelex-100 | Seawater | GFAAS and FAAS | [10] |
| Cu | Chelex-100 | — | Development of a valve | [11] |
| Ce, Co, Eu, Fe, Gd, Mn, Y & Zn | Chelex-100 | Brines | $\gamma$ spectrometry detection | [12] |
| Transition metals & Pb | Chelex-100 | Seawater | GFAAS & ICP detection | [13] |
| Transition metals & Pb | Chelex-100 | Seawater | GFAAS detection | [14] |
| Cd | Muromac-A1 | Waters, CRMs | Minicolumn, FI-ICP | [15] |
| Cr, Ti, V, Fe & Al | Muromac-A1 | — | Microcolumn, FI-ICP | [16] |
| Cd, Zn, Cu, Mn, Pb, Fe & Cr | Muromac-A1 | Biological CRMs | FI-AAS | [17] |
| Fe, Zn, Cu, Ni & Cr | Muromac-A1 | Water | Online-ICP or ICP-MS detection | [18] |
| Transition metals & Pb | Metpac CC-1 | Seawater | Online ICP-MS detection | [19] |
| Al | 8-quinolinol/EDTA | — | Comparision of chelating agents immobilised on glass | [20] |
| Fe | 8 quinolinol | — | Minicolumn, chelator on glass. CL detection. | [21] |
| Cu | 8 quinolinol | Waters | Chelator on glass | [22] |
| Transition metals | 8 quinolinol | Inorganic salts | Chelator on silica gel | [23] |
| Cu, Co, Cd, Ni, Pb & Zn | 8 quinolinol | Waters | Chelator on glass | [24] |
| Cu | 8 quinolinol | — | Chelator on glass. FI-ISE detection | [25] |
| Cr | 8 quinolinol | Seawater | Complexation of analyte followed by adsorption to a macroporous resin | [26] |
| Cu | 8-quinolinol | Water | Chelator on glass. FI-AAS | [27] |
| Cr | Poly(hydroxamic acid) resin | Seawater | FI-AAS | [28] |
| Hg | Picolinic acid amide | River water | Styrene DVB beads impregnated with chelator | [29] |
| Ag, Au, Cu, Fe, Hg, Ni & Zn | Histidine | Water | Histidine attached to carboxyl of amberlite IRC50 | [30] |
| Cu, Cd, Pb, Zn, Ag, Co, Fe, In, Ti, V, Bi | Carboxymethylated polyethylenimine-poly-methylene polyphenyl-ene isocyanate | Sea water, bone | No swelling of the resin | [31] |
| Cu, Cd, Zn | Chelex-100 and AG-MP-I | Waters | FI-AAS | [32] |
| Various | EDTrA-Cellulose/$HSO_3$ oxine cellulose | | | [33] |
| Hg | Thiazole & thiazoline groups | Seawater | Elution with HCl and thiourea | [34] |
| Pb | Various | Water | Chelating agents on silica gel | [35) |
| Mo and W | Kelex-100 | Seawater | Catalytic current polarography detection | [36] |
| Transition metals | Various | — | Preparation and study of chelators on silica gel | [37] |
| Ca and Mg | Dowex A1 | Brine | Minicolumn. Spectrophotometric detection | [38] |
| Various | XAD-4 with 7-dode-cenyl-8-quinolinol | Seawater | GFAAS and FAAS detection | [39] |
| Ni, Cu, Pb & Cd | Resion 122 | Waters | Minicolumn, salicylic acid functional group | [40] |

*Table 3. Some applications of cation exchange resins.*

| Analyte | Exchange resin | Matrix | Comments | Reference |
|---|---|---|---|---|
| Rare earths | AG 50 WX 8 | Geological materials | ICP detection (off-line) | [43] |
| Rare earths | AG 50 WX 8 | Geological materials | ICP detection | [44] |
| Li, Na, K, Ti, Be, Mg, Ni, Ca & Al | AG 50 WX 8 | Manganese | AAS detection | [45] |
| Rare earths | AG 50 WX 8 | Geological materials | ICP detection | [46] |
| Pt | AG 50 WX 8 | Airborne particulates | FI-ICP-MS detection | [47] |
| Mo | AG 50 WX 8 | Plants | Interferents adsorbed by resin—Mo not | [48] |
| Te | AG 50 WX 8 | — | HCl-acetone mobile phase elutes interferents | [49] |
| Rare earths | AG 50 WX 8 | Rocks | ICP detection | [50] |
| Rare earths | AG 50 WX 8/ AG 50 WX 12 | Geological | ICP detection | [51] |
| Rare earths | AG 50 WX 8/ AG 50 WX 12 | Geological | ICP detection | [52] |
| Mn, Co & Ni | AG MP 50 | Te | HCl-acetone mobile phase | [53] |
| Mn | Amberlite CG-120 | Ti, Si, HF, HCl, & $HNO_3$ | Spectrophotometric detection | [54] |
| Rare earths | Amberlite XAD-7 with PC-88A | Tb | Reduction of interferences | [55] |
| Eu, Th, U & Pu | Amberlyst A-15 | — | Extraction of metals from non-aqueous solutions | [56] |

*Table 4. Applications of anion exchange resins.*

| Analyte | Exchange medium | Matrix | Comments | Reference |
|---|---|---|---|---|
| Mn, Cu, Co, Zn, Cd, Pb & U | Dowex 1 × 8 | Mn nodules | Elution with either 6M HCl 1M HCl or 2M $HNO_3$ AAS detection | [57] |
| Ni | Dowex 1 × 8 | Mn nodules | Ni elutes with ethanol/HCl mobile phase. AAS detection | [58] |
| Ga | Dowex 1 × 8 | Mn nodules | AAS detection | [59] |
| Co | Dowex 1 × 8 | Water | GFAAS detection | [60] |
| Tl, Mo & V | Dowex 1 × 8 | Mn nodules | Chloro-complexes sorbed from sample | [61] |
| Th and U | Dowex 1 × 8 | Mn nodules | Fluorimetric detection | [62] |
| Zn | Dowex 1 × 8 | Plants | Zinc chloro complexes retained. Spectrophotmetric detection | [63] |
| Pt & Pd | Dowex 1 × 8 | Geological samples | GFAAS detection | [64] |
| Mn, Mg, Ti, Cr, W, Mo, Ta & Fe | Dowex AG 1 × 8 | Niobium | IC-ICP | [65] |
| Pt & Ir | AG 1 × 2 | Marine waters, sediments & organisms | GFAAS detection IC-ICP | [66] |
| Cd, Cr, Cu, Fe, Mn, Ni, Pb & Zn | AG MP 1 | River and seawater | ISOX complex adsorbed | [67] |
| B | IRA 743 | Water | Spectrophotometric detection | [68] |
| Zn & Cd | IRA 400 | — | CL detection | [69] |
| Al | IRA 400 | Biological samples | Microcolumn ICP or AAS detection | [70] |
| Ca | IRA 400 and De-acidite FF | — | Removal of phosphate and Sulphate interferences | [71] |

### Cation exchange

Cation exchangers have also been used for pre-concentration and matrix removal prior to atomic spectrometric detection. The most common cation exchange resins are AG 50W and Amberlite 120, both of which contain sulphonic acid functional groups. Recent applications of cation exchange resins are detailed in table 3. The eluent for cation exchange chromatography ranges from dilute acids to 8M nitric and 6M hydrochloric acids, depending upon the analyte. Further dilution may therefore be required before analysis by atomic spectrometry.

### Anion exchange

Anion exchange has also been used to facilitate analyses with atomic spectrometric detection. Anion exchange resins, such as Dowex 1 and Amberlite IRA 400, contain

*Table 5. Other exchange media used.*

| Analyte | Exchange medium | Matrix | Comments | Reference |
|---|---|---|---|---|
| Pb | Activated alumina | Water | FI-AAS microcolumn | [72] |
| Cr | Activated alumina | Urine | FI-ICP, minicolumn | [73] |
| S | Activated alumina | Water | FI-ICP, micro-column | [74] |
| Lanthanides | Naβaluminogallate crystals | — | Na exchange with lanthanides | [75] |
| Cr | C-18 Rainin column | — | Ion pairing of Cr VI with tetrabutylammonium phosphate | [76] |
| Various | Retardion II A 8 | — | Amphoteric resin | [77] |
| Se | Sephadex G-25M | Serum | Column used to de-salt serum ICP-MS detection | [78] |
| Cu, Pb | C-18 | — | Chelate formed with DDC, 8HQ PAR or PAN | [79] |

quaternary ammonium functional groups. Anion exchange can be used to quantitatively retain complexes of analytes with negatively charged ligands, whilst allowing other interfering cations to elute. Table 4 details some applications of anion exchange chromatography. In addition to retaining complexes of the analytes, there have been other applications. Kamson and Townshend [71] used an Amberlite IRA 400 and a De-acidite FF column to remove the interference effects exerted by phosphate and sulphate on calcium determinations by flame AAS.

### Others

Table 5 shows some novel methods of ion exchange. Activated alumina has the advantage of being amphoteric, i.e. in its basic form it may be used to adsorb cations, such as lead [72] and chromium [73]; and in its acidic form it may be used to adsorb anions, such as sulphate [74]. Similarly, Retardion 11A8 is an amphoteric resin with both benzyltrimethylammonium and carboxylic acid exchange groups. Non-polar C-18 (octadecylsilane) columns have also been used for some applications, such as the adsorption of the diethyldithiocarbamate complexes of copper and iron [79].

In addition to the papers detailing specific applications of FI and LC, there have been a number of authoritative reviews, for example on FI-ICP [80–83] and FI-atomic spectrometry [84–86]. A very comprehensive review of the literature is the second edition of Ruzicka and Hansen's book [87].

Several other papers concerned with FI-ICP or FI-ICP-MS also exist [88–95], however many were performed off-line [93–94].

### Conclusions

There is a growing trend towards the use of on-line mini- or micro-columns of exchange resins to pre-concentrate the analyte, or remove interfering species from the matrix, prior to atomic spectrometric detection. This may, in part, be due to the simplicity of the apparatus, and to the ease of automation of such systems. The use of on-line columns also leads to a decrease in the chances of contamination associated with the sample handling of off-line batch methods.

### Acknowledgements

The authors would like to acknowledge the funding of this work by The Ministry of Agriculture, Fisheries and Food.

### References

1. Riley, J. P. and Taylor, D., *Analytica Chimica Acta,* **40**, (1968), 479.
2. Fassett, J. D. and Kingston, H. M., *Analytical Chemistry,* **57** (1985), 2474.
3. Hartenstein, S. D., Ruzicka, J. and Christian, G. D., *Analytical Chemistry,* **57** (1985), 21.
4. Kayasth, S. R., Desai, H. B. and Sundaresan, M., *Analytica Chimica Acta,* **219** (1989), 313.
5. Kingston, H. M., Barnes, I. L., Brady, T. J., Rains, T. C. and Champ, M. A. *Analytical Chemistry,* **50** (1978), 2064.
6. Liu, Y. and Ingle, J. D., *Analytical Chemistry,* **61** (1989), 520.
7. Olsen, S., *Dansk Kemi,* **3** (1983), 68.
8. Olsen, S., Pessenda, L.C.R., Ruzicka, J. and Hansen, E. H., *Analyst,* **108** (1983), 905.
9. Paulson, A. J., *Analytical Chemistry,* **58** (1986), 183.
10. Rasmussen, L., *Analytica Chimica Acta,* **125** (1981), 117.
11. Jorgensen, S. S., Petersen, K. M. and Hansen, E. A., *Analytica Chimica Acta,* **169** [1985], 51.
12. Strachan, D. M., Tymochowicz, S., Schubert, P. and Kingston, H. M., *Analytica Chimica Acta,* **220** (1989), 243.
13. Sturgeon, R. E., Berman, S. S., Desaulniers, J. A. H., Mykytiuk, A. P., McClaren, J. W. and Russell, D. S., *Analytical Chemistry,* **52** (1980), 1585.
14. Su-Cheng Pai, Tien-Hsi Fang, Cheng-Tung A. Chen and Kwung Lung Jeng, *Marine Chemistry,* **29** (1990), 295.
15. Kumamaru, T., Matsuo, H., Okamoto, Y. and Ikeda, M., *Analytica Chimica Acta,* **181** (1986), 271.
16. Hirata, S., Umezaki, Y. and Ikeda, M., *Analytical Chemistry,* **58** (1986), 2602.
17. Hirata, S., Honda, K. and Kumamaru, T., *Analytical Chimica Acta,* **221** (1989), 65.
18. Ida, I., Yoshikawa, H., Ishibashi, Y. and Gunji, N., *ICP Inf. Newsl.,* **16** (1990), 388.
19. James, D. L., The Specialist, Thermo Electron Ltd (13 September 1990).
20. Allen, E. A., Boardman, M. C. and Plunkett, B. A., *Analytica Chimica Acta,* **196** (1987), 323.

21. Alwarthan, A. A., Habib, K. A. and Towshend, A., *Fresenius J. Analytical Chemistry,* **337** (1990), 848.
22. Guedes Da Mota, M. M., Romer, F. G. and Griepink, B., *Fresenius Z. Analytical Chemistry,* **287** (1977), 19.
23. Hill, J. M., *Journal of Chromatography,* **76** (1973), 455.
24. Malamas, F., Bengtsson, M. and Johansson, G., *Analytica Chimica Acta,* **160** (1984), 1.
25. Risinger, L., *Analytica Chimica Acta,* **179** (1986), 509.
26. Isshiki, K., Sohrin, Y., Karatani, H. and Nakayama, E., *Analytica Chimica Acta,* **224** (1989), 55.
27. Marshall, M. A. and Mottola, H. A., *Analytical Chemistry,* **57** (1985), 729.
28. Shah, A. and Devi, S., *Analytica Chimica Acta,* **236** (1990), 469.
29. Sengupta, B. and Das, J., *Analytica Chimica Acta,* **219** (1989), 339.
30. Liu, C. Y., *Analytica Chimica Acta,* **192** (1987), 85.
31. Horvath, Z. and Barnes, R. M., *Analytical Chemistry,* **58** (1986), 1352.
32. Liu, Y. and Ingle, J. D., *Analytical Chemistry,* **61** (1989), 525.
33. Prakash, N., Csanady, G., Michaelis, M. R. A. and Knapp, G., *Mikrochimica Acta* (1989), 257.
34. Sugii, A., Ogawa, N. and Hashizume, H., Talanata, **27** (1980), 627.
35. Bysouth, S. R., Tyson, J. F. and Stockwell, P. B., *Analytica Chimica Acta,* **214** (1988), 329.
36. Sohrin, Y., Isshiki, K., Nakayama, E., Kihara, S. and Matsui, M., *Analytica Chimica Acta,* **218** (1989), 25.
37. Leyden, D. E. and Luttrell, G. H., *Analytical Chemistry,* **47** (1975), 1612.
38. Wada, H., Asakura, K., Rattaiah, G. V. and Nakagawa, G., *Analytica Chimica Acta,* **214** (1988), 439.
39. Isshiki, K., Tsuji, F., Kuwamoto, T., and Nakayama, E., *Analytical Chemistry,* **59** (1987), 2491.
40. Fang, Z., Xu, S. and Zhang, S., *Analytica Chimica Acta,* **164** (1984), 41.
41. Handley, H. (Personal communication).
42. Jiang, S. J., Palmieri, M. D., Fritz, J. S. and Houk, R. S., *Analytica Chimica Acta,* **200** (1987), 559.
43. Crock, J. G., Lichte, F. E. and Wildeman, T. R., *Chem. Geol.,* **45** (1984), 149.
44. Crock, J. G., Lichte, F. E., Riddle, G. O. and Beech, C. L., *Talanta,* **33** (1986), 601.
45. Faisca, A. M. M. M., Victor, A. H. and Bohmer, R. G., *Analytica Chimica Acta,* **215** (1988), 111.
46. Iwasaki, K and Haraguchi, H., *Analytica Chimica Acta,* **208** (1988), 163.
47. Mukai, H., Ambe, Y. and Morita, M., *Journal of Analytical Atomic Spectrometry,* **5** (1990), 75.
48. Pessenda, L. C. R., Jacintho, A. O. and Zagatto, E. A. G., *Analytica Chimica Acta,* **214** (1988), 239.
49. Strelow, F. W. E., *Analytical Chemistry,* **56** (1984), 2069.
50. Walsh, J. N., Buckley, F. and Barker, J., *Chem. Geol.,* **33** (1981), 141.
51. Webster, J. R. and Gilstrap, M. S., *Chem. Geol.,* **85** (1990), 287.
52. Zachmann, D. W., *Analytical Chemistry,* **60** (1988), 420.
53. Strelow, F. W. E., *Analytica Chimica Acta,* **212** (1988), 191.
54. Zhang, C., Kawakubo, S. and Fukasawa, T., *Analytica Chimica Acta,* **217** (1989), 23.
55. Kobayashi, S. and Suzuki, T. M., *ICP Inform. Newsl.,* **16,** (1990), 388.
56. Achuthan, P. V., Jangida, B. L. and Sundaresan, M., J. *Rad. Nucl. Chem.,* **141** (1990), 171.
57. Korkisch, J., Hubner, H., Steffan, I., Arrhenius, G.,Fisk, M and Frazer, J., *Analytica Chimica Acta,* **83** (1976), 83.
58. Korkisch, J., Steffan, I. and Fisk, M. B., *Analytica Chimica Acta,* **108** (1979), 63.
59. Korkisch, J., Steffan, I., Nonaka, J. and Arrhenius, G., *Analytica Chimica Acta,* **109** (1979), 181.
60. Stella, R., Ganzerli Valentini, M. T. and Maggi, L., *Analytical Chemistry,* **57** (1985), 1941.
61. Korkisch, J., Steffan I. and Arrhenius, G., *Analytica Chimica Acta,* **94** (1977), 237.
62. Korkisch, J., Steffan, I., Arrhenius, G., Fisk, M. and Frazer, J., *Analytica Chimica Acta,* **90** (1977), 151.
63. Ferreira, J. R., Zagatto, E. A. G., Arruda, M. A. Z. and Brienza, S. M. B., *Analyst,* **115** (1990), 779.
64. Branch, C. H., and Hutchison, D., *Journal of Analytical Atomic Spectrometry,* **1** (1986), 433.
65. Imakita, T., Fudagawa, N. and Kubota, M., *Analyst,* **115** (1990), 1185.
66. Hodge, V., Stallard, M., Koide, M., Goldberg, E. D., *Analytical Chemistry,* **58** (1986), 616.
67. Porta, V., Sarzanini, C. and Mentasti, E., *Mikrochim. Acta,* (1989), 247.
68. Sekerka, I. and Lechner, J. F., *Analytica Chimica Acta,* **234** (1990), 199.
69. Burguera, J. L., Burguera, M. and Townshend, A., *Analytica Chimica Acta,* **127** (1981), 199.
70. Pereiro Garcia, M. R., Diaz Garcia, M. E. and Sanz Medel, A., *Journal of Analytical Atomic Spectrometry,* **2** (1987), 699.
71. Kamson, O. F. and Townshend, A., *Analytica Chimica Acta,* **155** (1983), 253.
72. Zhang, Y., Riby, P., Cox, A. G., McCleod, C. W., Date, A. R. and Cheung, Y. Y., *Analyst,* **113** (1988), 125.
73. Cox, A. G. and McCleod, C. W., *Analytica Chimica Acta,* **179** (1986), 487.
74. Cox, A. G., McCleod, C. W., Miles, D. L. and Cook, J. M., *Journal of Analytical Atomic Spectrometry,* **2** (1987), 553.
75. Aka, G., Thery, J., and Vivien, D., *Solid State Ionics,* **39** (1990), 225.
76. Syty, A., Christensen, R. G. and Rains, T. C., *Atomic Spectroscopy,* **7** (1986), 89.
77. Aldabbgh, S. S. and Dybczynski, R., *J. Rad. Nuc. Chem.,* **92** (1985), 37.
78. Lyon, T. D. B., Fell, G. S., Hutton, R. C. and Eaton, A. N., *Journal of Analytical Atomic Spectrometry,* **3** (1988), 601.
79. Ruzicka, J. and Arndal, A., *Analytica Chimica Acta,* **216** (1989), 243.
80. Christian, G. D. and Ruzicka, J., *Spectrochim. Acta,* **42B** (1987), 157.
81. McCleod, C. W., *Journal of Analytical Atomic Spectrometry,* **2** (1987), 549.
82. Barnes, R. M., *Spectroscopy,* **1** (1986), 24.
83. Christian, G. D., and Ruzicka, J., *Spectrochim. Acta,* Part B, **42** (1987), 157.
84. Tyson, J. F., *Analytica Chimica Acta,* **214** (1988), 57.
85. Puchades, R., Maquieira, A., Atienza, J. and Herrero, M. A., *Journal of Automatic Chemistry,* **12** (1990), 163.
86. Tyson, J. F., *Analytica Chimica Acta,* **234** (1990), 3.
87. Ruzicka, J. and Hansen, E. H., *Flow Injection Analysis,* 2nd edn (Wiley, New York).
88. McCarthy, J. A., Caruso, J. A. and Fricke, F. L., *Journal of Chromatographic Science,* **21** (1983), 389.
89. Bushee, D., Krull, I. S., Savage, R. N. and Smith, S. B., *Journal of Liquid Chromatography,* **5** (1982), 463.
90. Lawrence, K. E., Rice, G. W. and Fassel, V. A., *Analytical Chemistry,* **56** (1984), 289.

91. Hausler, D. W. and Taylor, L. T., *Analytical Chemistry,* **53** (1981), 1223.
92. Irgolic, K. J., Stockton, R. A. and Chakraborti, D., *Spectrochimica Acta,* **38B** (1983), 437.
93. Aulis, R., Bolton, A., Doherty, W., Vander Voet, A. and Wang, P., *Spectrochimica Acta,* **40B** 1985, 377.
94. McClaren, J. W., Mykytiuk, A. P., Willie, S. N. and Berman, S. S., *Analytical Chemistry,* **57** (1985), 2907.
95. Doherty, W. and Vander Voet, A., *Canadian Journal of Spectroscopy,* **30** (1986), 135.
96. Munro, S., Ebdon, L. and McWeeny, D. J., *Journal of Analytical Atomic Spectrometry,* **1** (1986), 211.

